# Supplementary material for: Clock-like Mutation Signature May Be Prognostic for Worse Survival Than Signatures of UV Damage in Cutaneous Melanoma
Source: Cancers (Basel). 2023 Jul 27;15(15):3818. doi: 10.3390/cancers15153818 (PMC10418148; doi:10.3390/cancers15153818)
Supplement: Supplementary file 1 [file cancers-15-03818-s001.zip › cancers-2421654-supplementary.pdf]

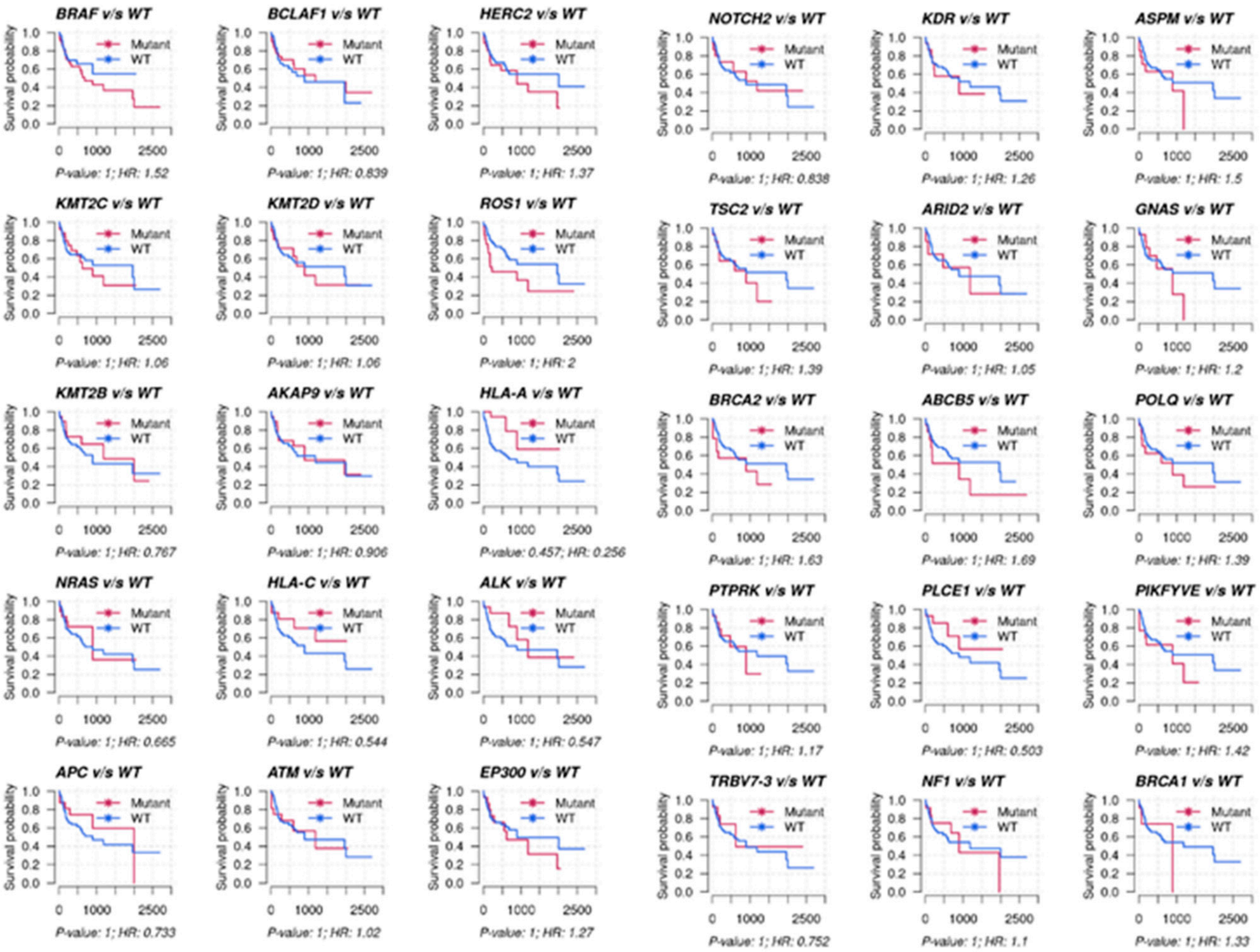

Figure S1. Survivorship analyses for single gene mutations in the whole study cohort. The most frequently mutated genes, affected in at least 14 patients. Read from left to right.
